# Reply to Drs. Angulo and Collignon

**DOI:** 10.3201/eid0604.000430

**Published:** 2000

**Authors:** Margaret A. Davis, Dale D. Hancock, Thomas E. Besser, Daniel H. Rice, John M. Gay

**Affiliations:** Washington State University, Pullman, Washington, USA

**Keywords:** antimicrobial drug resistance, tetracycline, MR-DT104, Campylobacter, methicillin-resistant Staphylococcus aureus, MRSA


**Frederick J. Angulo and Patricia M. Griffin**
Centers for Disease Control and Prevention,
Atlanta, Georgia, USA

### References

1. Davis MA, Hancock DD, Besser TE, Rice DH, Gay JM, Gay C, et. al. Changes in antimicrobial resistance among *Salmonella enterica* serovar Typhimurium isolates from humans and cattle in the Northwestern United States, 1982-1997. Emerg Infect Dis 1999;5:802-6.
2. Sandvang D, Aarestrup FM, Jensen LB. Characterisation of integrons and antibiotic resistance genes in Danish multiresistant *Salmonella enterica* Typhimurium DT104. FEMS Microbiol Let 1998;160:37-41.
3. Ridley A, Threlfall EJ. Molecular epidemiology of antibiotic resistance genes in multiresistant epidemic *Salmonella typhimurium* DT104. Microb Drug Resist 1998;4:113-8.
4. Briggs CE, Fratamico PM. Molecular characterization of an antibiotic resistance gene cluster of *Salmonella typhimurium* DT104. Antimicrob Agents Chemother 1999;43:846-9.
5. Bolton LF, Kelly LC, Lee MD, Fedorka-Cray PI, Maurer H. Detection of multidrug-resistant *Salmonella enterica* serotype *typhimurium* DT104 based on a gene which confers cross-resistance to florfenicol and chloramphenicol. J Clin Microbiol 1999;37:1348-51.
6. Zhao J, Aoki T. Nucleotide sequence analysis of the class G tetracycline resistance determinant from *Vibrio anguillarum*. Microbiol Immunol 1992;36:1051-60.
7. Kim EH, Aoki T. Drug resistance and broad geographical distribution of identical R plasmids of *Pasteurella piscicida* isolated from cultured yellowtail in Japan. Microbiol Immunol 1993;37:103-9.
8. Cohen ML, Tauxe RV. Drug-resistant *Salmonella* in the United States: an epidemiologic perspective. Science 1986;234:964-9.
9. Clark GM, Kaufmann AF, Gangrosa EJ. Epidemiology of an international outbreak of *Salmonella agona*. Lancet 1973;490-3.
10. Centers for Veterinary Medicine, U.S. Food & Drug Administration. Proposed framework for evaluating and assuring the human safety of the microbial effects of antimicrobial new animal drugs intended for use in food-producing animals. Washington: FDA; 1999 Jan 6. Available from: URL:http//www.fda.gov/cvm/fda/infores/vmac/antim18.htm

### Reply to Drs. Angulo and Collignon

**To the Editor:** Drs. Angulo and Collignon point out that exposure to one antimicrobial drug (e.g., tetracycline) can confer a selective advantage to a multiresistant organism (e.g., R-type ACSSuT) over nonresistant organisms. However, tetracycline use would not be expected to favor one tetracycline-resistant organism (MR-DT104) over other tetracycline-resistant organisms, and most bovine Typhimuriums before the MR-DT104 epidemic were tetracycline-resistant R-type ASSuT. Since neither florfenicol nor chloramphenicol was then available for use in livestock in the United States, the evidence suggests that the emergence of MR-DT104 in cattle populations was not driven by antibiotic selection pressure. The references Drs. Angulo and Collignon cited to establish the importance of antimicrobial use in livestock for the dissemination of multiresistant clones either do not address the issue of dissemination (1-2) or present evidence to the contrary: the dissemination of fluoroquinolone-resistant MR-DT104 despite the lack of fluoroquinolone use in the herds in question (3).

Available data support Dr. Collignon's example of *Campylobacter* as an agent for which fluoroquinolone use in livestock resulted in increasing prevalence of fluoroquinolone resistance (4). However, the epidemiology of resistance in polyclonal commensals such as *Campylobacter* is very unlike that of epidemic, clonal *S.* Typhimuriums. The epidemic, clonal dissemination of *S.* Typhimurium more closely resembles that of methicillin-resistant *Staphylococcus aureus* (MRSA). Epidemic MRSA clones differ genetically from nonepidemic ones, and dissemination of epidemic clones does not necessarily require antimicrobial selection pressure (5). Because antimicrobial usage practices that contribute to the control of MRSA have not been scientifically defined, infection control practices must play the central role in successful MRSA control programs (6-8).

Dr. Angulo's hypothesis that MR-DT104 emerged genetically in Asian fish is plausible, but other credible hypotheses exist. *tet*(G), first described in *Vibrio angullarium*, also occurs in *Pseudomonas aeruginosa* (9). Similarly, *flo*R is closely related to the *P. aeruginosa* chloramphenicol-resistance gene *cmlA* (10), and *pse*-1 encoded beta-lactamase is a common feature of hospital *P. aeruginosa* isolates (11). Thus, the hypothesis that MR-DT104 acquired resistance genes horizontally from nosocomial pseudomonads might also be worthy of consideration.

*Salmonella* infections acquired in other countries are frequently diagnosed in travelers recently returned to the United States. Although transmission of these infections to other humans may be rare in the United States, human-to-bovine transmission may occur regularly: thousands of cases of bovine *Taenia saginata* cysticercosis occurred immediately before and during the dissemination of MR-DT104. As humans are the only definitive host of the *T. saginata* tapeworm, these cases confirm the large-scale occurrence of human-to-animal transmission of enteropathogenic agents in the United States. Since transmission of *S.* Typhimurium from herd to herd is common in the United States, increased emphasis on *Salmonella* infection control may be an effective method for reducing dissemination of organisms such as MR-DT104.

With or without imposition of stringent controls on antibiotic use in the United States and Europe, the future genetic emergence of new epidemic clones of *S.* Typhimurium somewhere in the world is highly likely, and controlling the dissemination of epidemic clones is essential to avoid increasing problems with multidrug resistance. Certainly we do not disagree with the concept of reducing antimicrobial use, particularly such frivolous use as in calf milk replacers. However, we urge public health officials to consider that infection control is as central to control of agents such as MR-DT104 as it is for epidemic MRSA.

**Margaret A. Davis, Dale D. Hancock, Thomas E. Besser, Daniel H. Rice, John M. Gay**
Washington State University, Pullman, Washington, USA

### References

1. Cohen ML, Tauxe RV. Drug-resistant *Salmonella* in the United States: an epidemiologic perspective. Science 1986;234:964-9.
2. Glynn MK, Bopp C, Dewitt MS, Dabney P, Mokhtar M, Angulo FJ. Emergence of multidrug-resistant *Salmonella enterica* serotype Typhimurium DT104 infections in the United States. N Engl J Med 1998;338:1333-8.
3. Mølbak K, Baggesen DL, Aarestrup FM, Ebbesen JM, Engberg J, Frydendahl K, et al. An outbreak of multidrug-resistant, quinolone-resistant *Salmonella enterica* serotype Typhimurium DT104. N Engl J Med 1999;341:1420-5.
4. Smith KE, Besser JM, Hedberg CW, Leano FT, Bender JB, Wicklund JH, et al. Quinolone-resistant *Campylobacter jejuni* infections in Minnesota, 1992-1998. N Engl J Med 1999;340:1525-32.
5. Papakyriacou H, Vaz D, Simor A, Louie M, McGavin MJ. Molecular analysis of the accessory gene regulator (agr) locus and balance of virulence factor expression in epidemic methicillin-resistant *Staphylococcus aureus*. J Infect Dis 2000;181:990-1000.
6. Wagenvoort JHT. Dutch measures to control MRSA and the expanding European Union. Eurosurveillance 2000;5:26-8.
7. Lipsitch M, Bergstrom CT, Levin BR. The epidemiology of antibiotic resistance in hospitals. Proc Nat Acad Sci U S A 2000;97:1938-43.
8. McGowan JE Jr. Strategies for study of the role of cycling on antimicrobial use and resistance. Infect Control Hosp Epidemiol 2000 Jan;21(1 Suppl):S36-S43.
9. Ng LK, Mulvey MR, Martin I, Peters GA, Johnson W. Genetic characterization of antimicrobial resistance in Canadian isolates of *Salmonella* Serovar Typhimurium DT104. Antimicrob Agents Chemother 1999;43:3018-21.
10. Briggs CE, Fratamico PM. Molecular characterization of an antibiotic resistance gene cluster of *Salmonella typhimurium* DT104. Antimicrob Agents Chemother 1999;43:846-9.
11. Pitt TL, Livermore DM, Miller G, Vatopoulos A, Legakis NJ Resistance mechanisms of multiresistant serotype 012 *Pseudomonas aeruginosa* isolated in Europe. J Antimicrob Chemother 1990;26(3):319-28.

### Malaria and Global Warming in Perspective?

**To the Editor:** The two reports from the International Panel on Climate Change (IPCC) (1,2) cited in the letter by Pim Martens (3) are widely regarded as “the standard scientific reference for all concerned with climate change and its consequences,” yet the contents of these reports are often misleading. The quoted passage does not acknowledge the devastation caused by malaria in temperate regions. The reassurance that “existing public health resources” would “make reemergent malaria unlikely” ignores the nonclimatic factors that led to its disappearance and continued absence. Moreover, although malaria/climate models are not meant to predict future worlds, the IPCC chapter (1) on human health—one-third of which is devoted to vector-borne disease—makes extensive use of such models to warn of substantial “actual climate-related increases in malaria incidence” and “highly likely” exten-

**To the Editor:** Drs. Angulo and Collignon point out that exposure to one antimicrobial drug (e.g., tetracycline) can confer a selective advantage to a multiresistant organism (e.g., R-type ACSSuT) over nonresistant organisms. However, tetracycline use would not be expected to favor one tetracycline-resistant organism (MR-DT104) over other tetracycline-resistant organisms, and most bovine Typhimuriums before the MR-DT104 epidemic were tetracycline-resistant R-type ASSuT. Since neither florfenicol nor chloramphenicol was then available for use in livestock in the United States, the evidence suggests that the emergence of MR-DT104 in cattle populations was not driven by antibiotic selection pressure. The references Drs. Angulo and Collignon cited to establish the importance of antimicrobial use in livestock for the dissemination of multiresistant clones either do not address the issue of dissemination (1-2) or present evidence to the contrary: the dissemination of fluoroquinolone-resistant MR-DT104 despite the lack of fluoroquinolone use in the herds in question (3).

Available data support Dr. Collignon's example of *Campylobacter* as an agent for which fluoroquinolone use in livestock resulted in increasing prevalence of fluoroquinolone resistance (4). However, the epidemiology of resistance in polyclonal commensals such as *Campylobacter* is very unlike that of epidemic, clonal *S*. Typhimuriums. The epidemic, clonal dissemination of *S*. Typhimurium more closely resembles that of methicillin-resistant *Staphylococcus aureus* (MRSA). Epidemic MRSA clones differ genetically from nonepidemic ones, and dissemination of epidemic clones does not necessarily require antimicrobial selection pressure (5). Because antimicrobial usage practices that contribute to the control of MRSA have not been scientifically defined, infection control practices must play the central role in successful MRSA control programs (6-8).

Dr. Angulo's hypothesis that MR-DT104 emerged genetically in Asian fish is plausible, but other credible hypotheses exist. *tet*(G), first described in *Vibrio angullarium*, also occurs in *Pseudomonas aeruginosa* (9). Similarly, *flo*R is closely related to the *P. aeruginosa* chloramphenicol-resistance gene *cmlA* (10), and *pse*-1 encoded beta-lactamase is a common feature of hospital *P. aeruginosa* isolates (11). Thus, the hypothesis that MR-DT104 acquired resistance genes horizontally from nosocomial pseudomonads might also be worthy of consideration.

*Salmonella* infections acquired in other countries are frequently diagnosed in travelers recently returned to the United States. Although transmission of these infections to other humans may be rare in the United States, human-to-bovine transmission may occur regularly: thousands of cases of bovine *Taenia saginata* cysticercosis occurred immediately before and during the dissemination of MR-DT104. As humans are the only definitive host of the *T. saginata* tapeworm, these cases confirm the large-scale occurrence of human-to-animal transmission of enteropathogenic agents in the United States. Since transmission of *S*. Typhimurium from herd to herd is common in the United States, increased emphasis on Salmonella infection control may be an effective method for reducing dissemination of organisms such as MR-DT104.

With or without imposition of stringent controls on antibiotic use in the United States and Europe, the future genetic emergence of new epidemic clones of *S*. Typhimurium somewhere in the world is highly likely, and controlling the dissemination of epidemic clones is essential to avoid increasing problems with multidrug resistance. Certainly we do not disagree with the concept of reducing antimicrobial use, particularly such frivolous use as in calf milk replacers. However, we urge public health officials to consider that infection control is as central to control of agents such as MR-DT104 as it is for epidemic MRSA.
